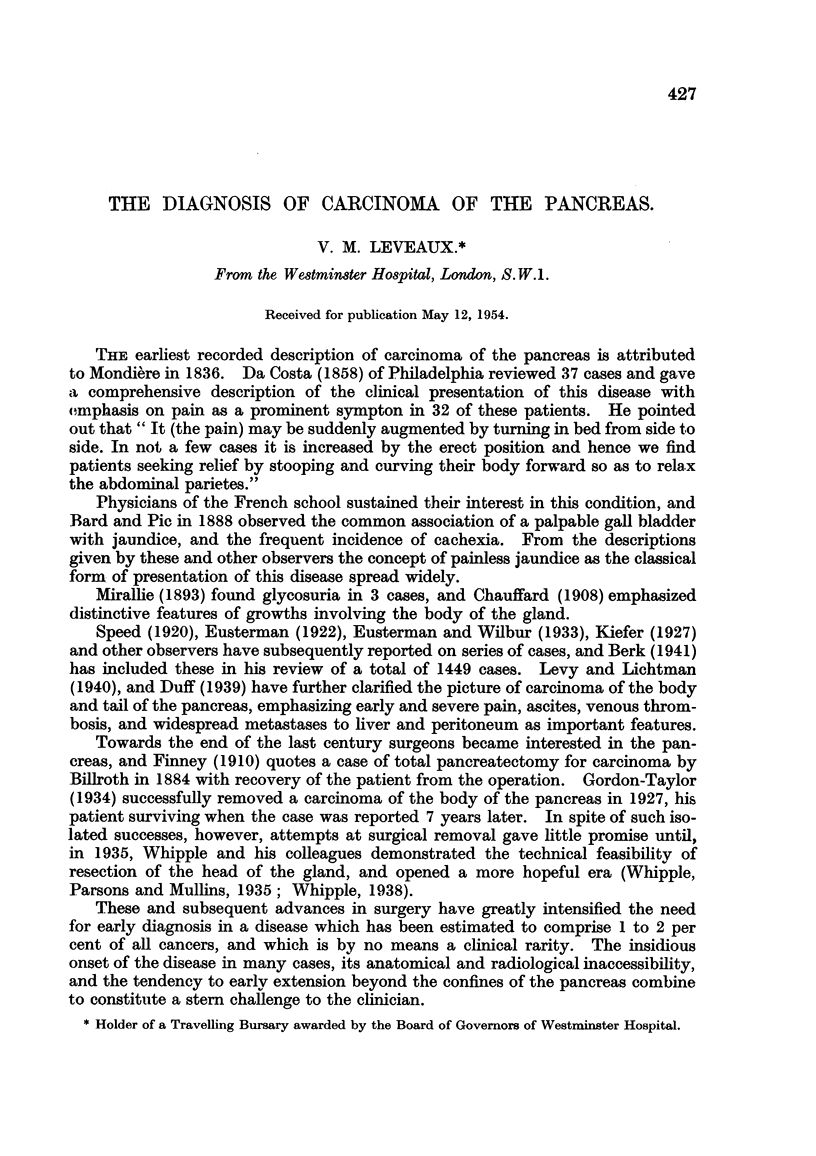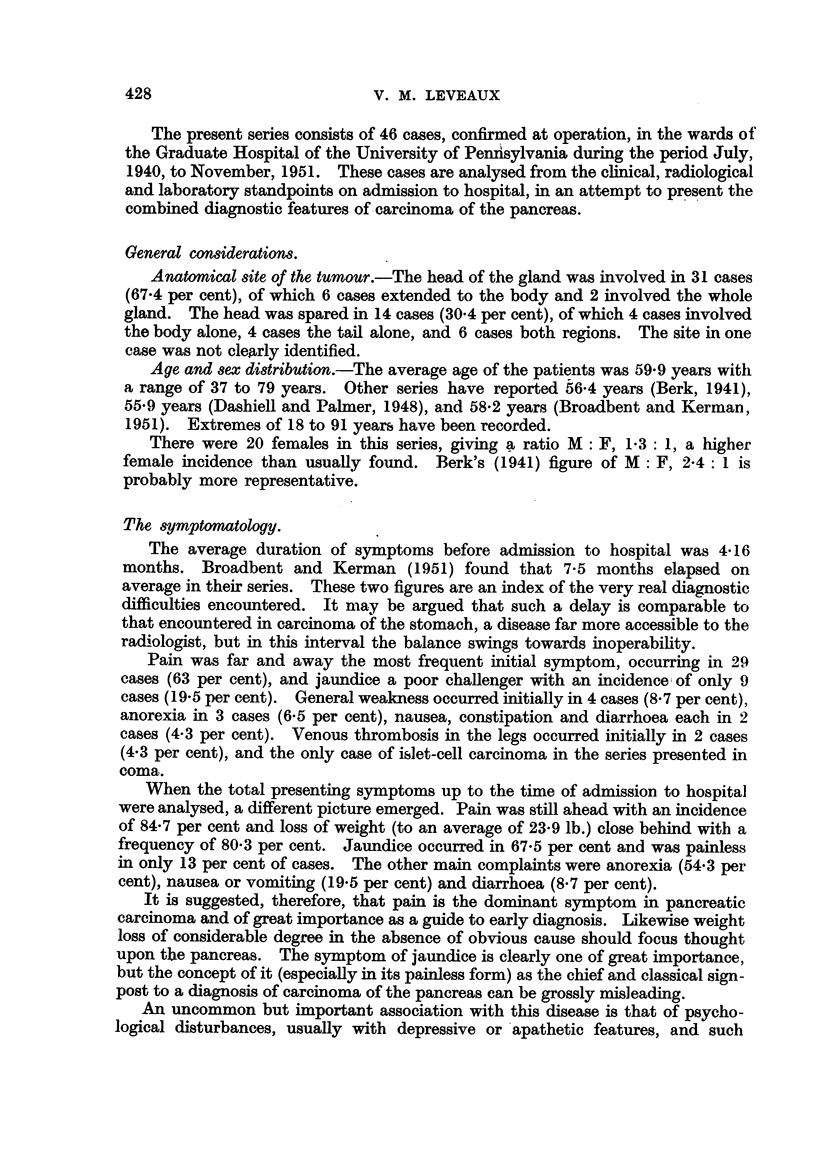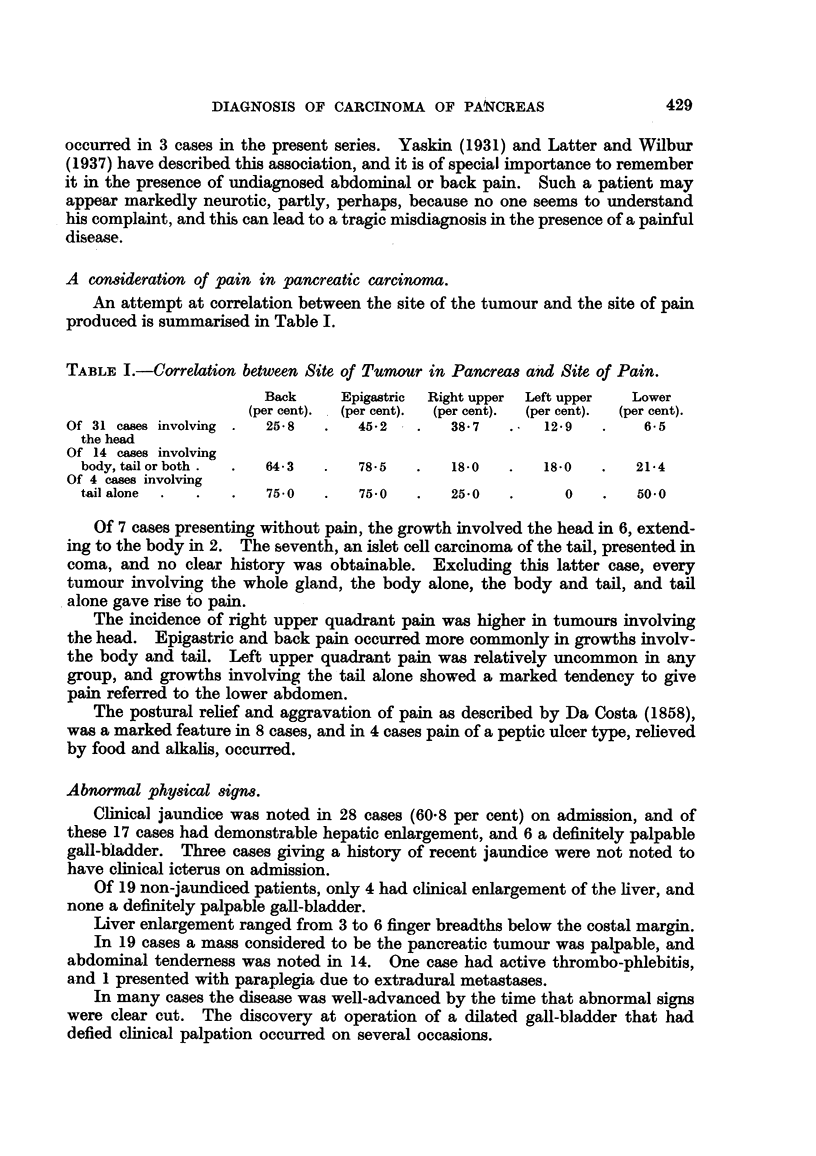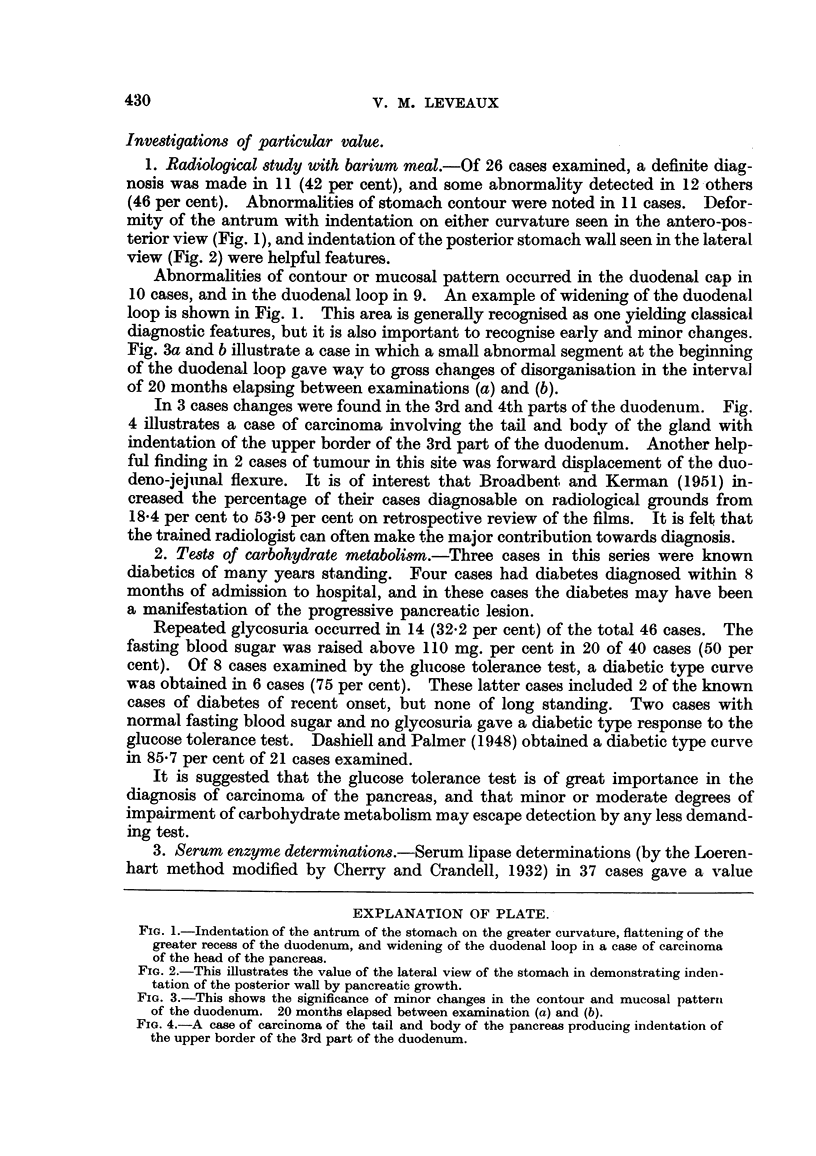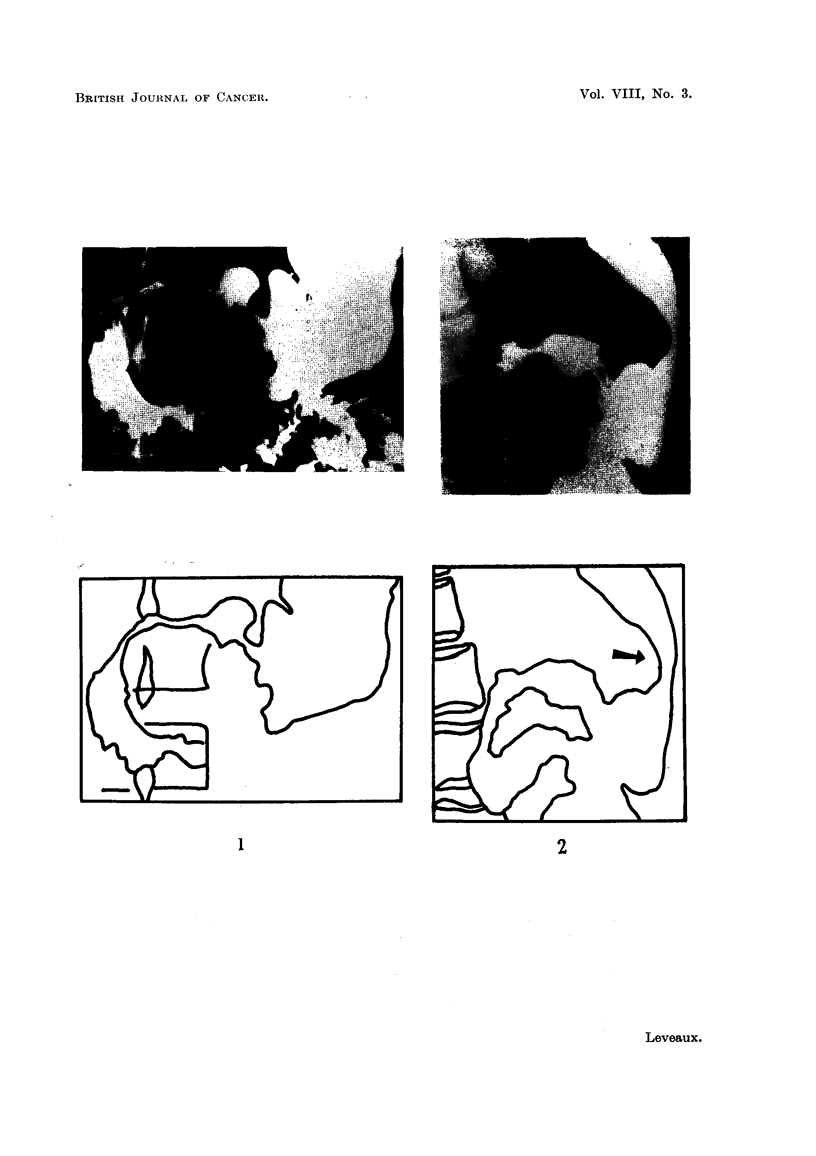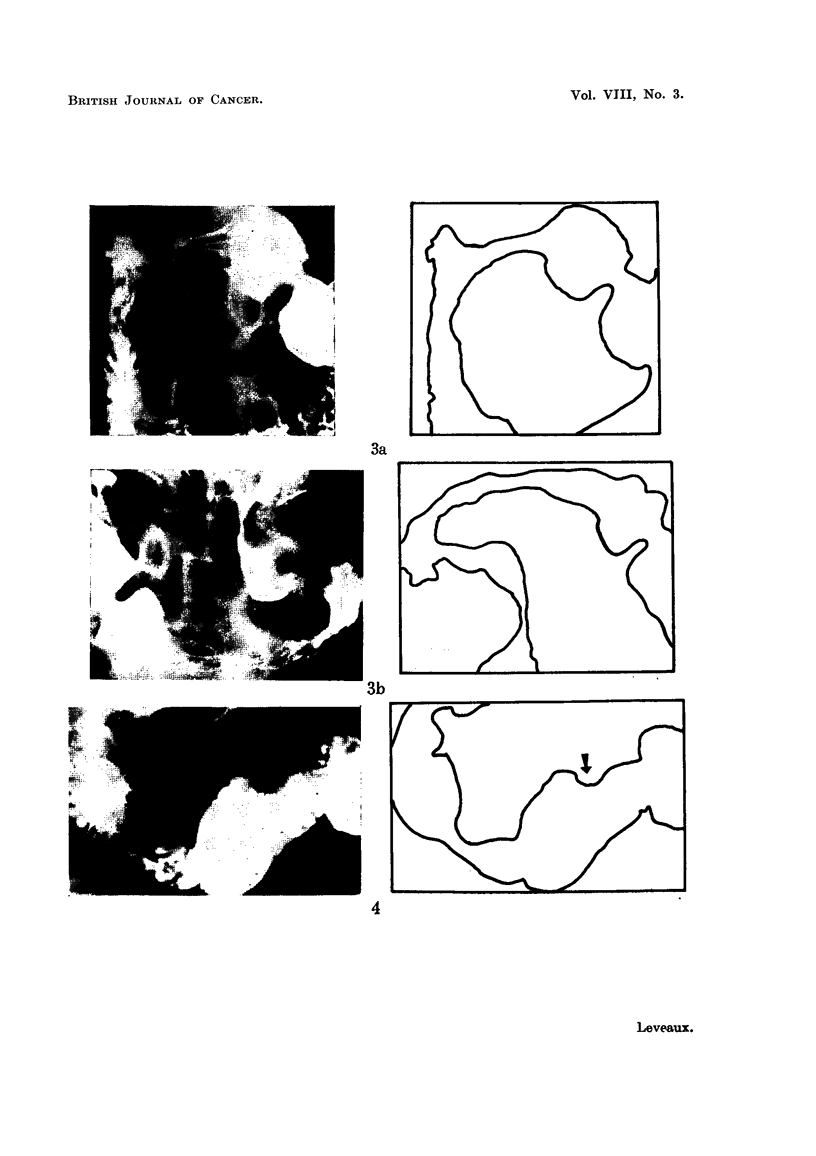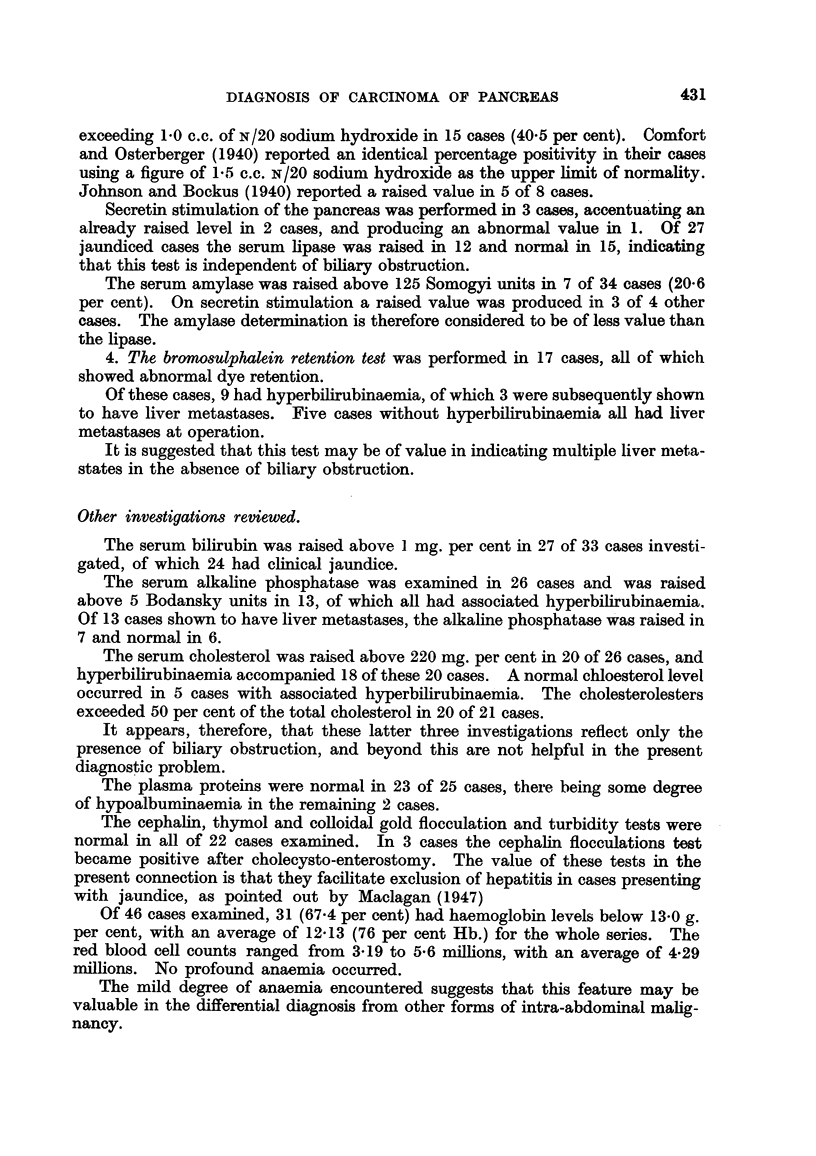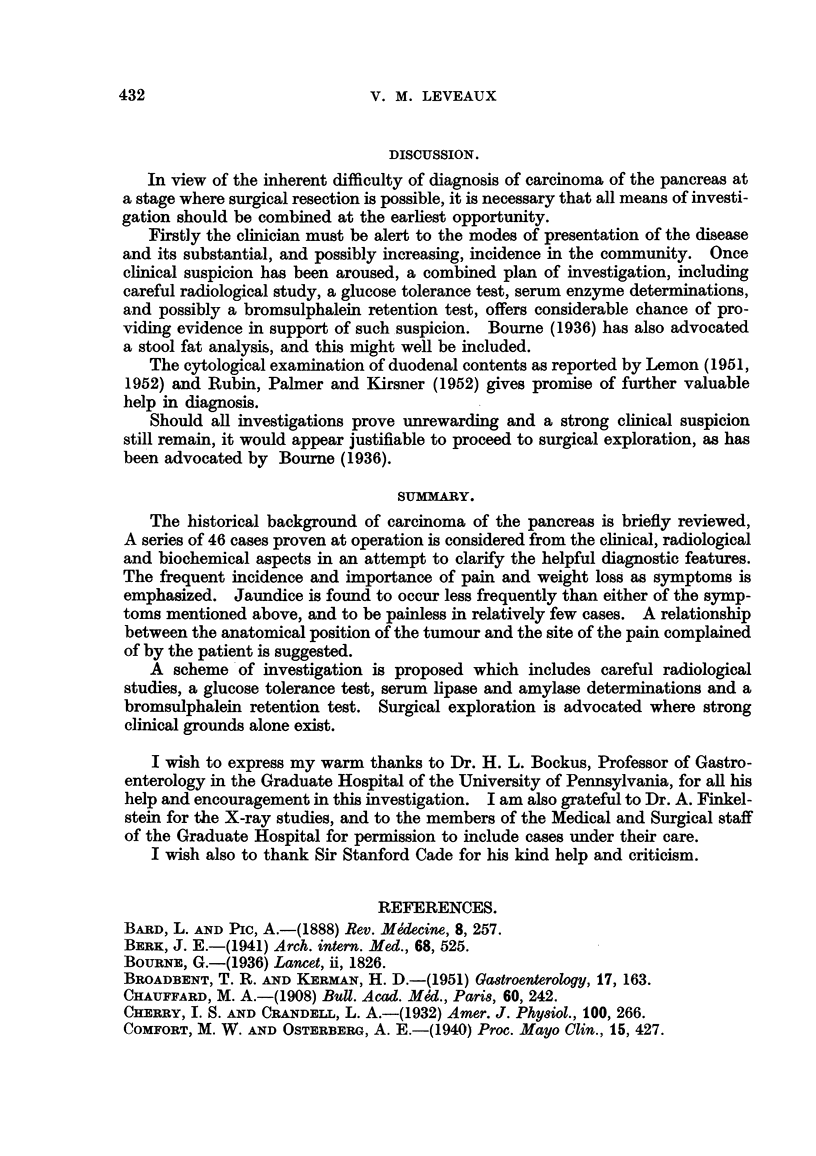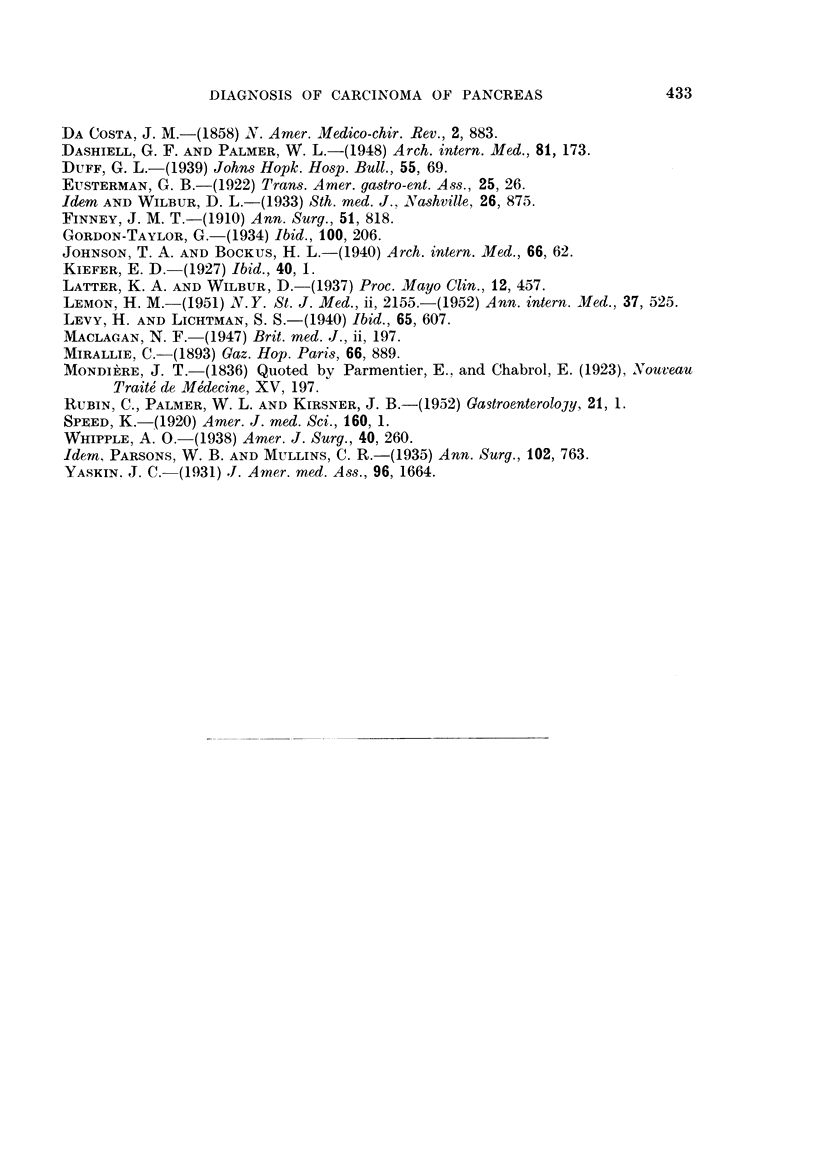# The Diagnosis of Carcinoma of the Pancreas

**DOI:** 10.1038/bjc.1954.45

**Published:** 1954-09

**Authors:** V. M. Leveaux

## Abstract

**Images:**


### THE DIAGNOSIS OF CARCINOMA OF THE PANCREAS.

V. M. LEVEAUX.*

*From the Westminster Hospital, London, S.W.1.*

Received for publication May 12, 1954.

THE earliest recorded description of carcinoma of the pancreas is attributed to Mondière in 1836. Da Costa (1858) of Philadelphia reviewed 37 cases and gave a comprehensive description of the clinical presentation of this disease with emphasis on pain as a prominent sympton in 32 of these patients. He pointed out that " It (the pain) may be suddenly augmented by turning in bed from side to side. In not a few cases it is increased by the erect position and hence we find patients seeking relief by stooping and curving their body forward so as to relax the abdominal parietes."

Physicians of the French school sustained their interest in this condition, and Bard and Pic in 1888 observed the common association of a palpable gall bladder with jaundice, and the frequent incidence of cachexia. From the descriptions given by these and other observers the concept of painless jaundice as the classical form of presentation of this disease spread widely.

Mirallie (1893) found glycosuria in 3 cases, and Chauffard (1908) emphasized distinctive features of growths involving the body of the gland.

Speed (1920), Eusterman (1922), Eusterman and Wilbur (1933), Kiefer (1927) and other observers have subsequently reported on series of cases, and Berk (1941) has included these in his review of a total of 1449 cases. Levy and Lichtman (1940), and Duff (1939) have further clarified the picture of carcinoma of the body and tail of the pancreas, emphasizing early and severe pain, ascites, venous thrombosis, and widespread metastases to liver and peritoneum as important features.

Towards the end of the last century surgeons became interested in the pancreas, and Finney (1910) quotes a case of total pancreatectomy for carcinoma by Billroth in 1884 with recovery of the patient from the operation. Gordon-Taylor (1934) successfully removed a carcinoma of the body of the pancreas in 1927, his patient surviving when the case was reported 7 years later. In spite of such isolated successes, however, attempts at surgical removal gave little promise until, in 1935, Whipple and his colleagues demonstrated the technical feasibility of resection of the head of the gland, and opened a more hopeful era (Whipple, Parsons and Mullins, 1935 ; Whipple, 1938).

These and subsequent advances in surgery have greatly intensified the need for early diagnosis in a disease which has been estimated to comprise 1 to 2 per cent of all cancers, and which is by no means a clinical rarity. The insidious onset of the disease in many cases, its anatomical and radiological inaccessibility, and the tendency to early extension beyond the confines of the pancreas combine to constitute a stern challenge to the clinician.

* Holder of a Travelling Bursary awarded by the Board of Governors of Westminster Hospital.

The present series consists of 46 cases, confirmed at operation, in the wards of the Graduate Hospital of the University of Pennsylvania during the period July, 1940, to November, 1951. These cases are analysed from the clinical, radiological and laboratory standpoints on admission to hospital, in an attempt to present the combined diagnostic features of carcinoma of the pancreas.

*General considerations.*

*Anatomical site of the tumour.*—The head of the gland was involved in 31 cases (67·4 per cent), of which 6 cases extended to the body and 2 involved the whole gland. The head was spared in 14 cases (30·4 per cent), of which 4 cases involved the body alone, 4 cases the tail alone, and 6 cases both regions. The site in one case was not clearly identified.

*Age and sex distribution.*—The average age of the patients was 59·9 years with a range of 37 to 79 years. Other series have reported 56·4 years (Berk, 1941), 55·9 years (Dashiell and Palmer, 1948), and 58·2 years (Broadbent and Kerman, 1951). Extremes of 18 to 91 years have been recorded.

There were 20 females in this series, giving a ratio M : F, 1·3 : 1, a higher female incidence than usually found. Berk's (1941) figure of M : F, 2·4 : 1 is probably more representative.

*The symptomatology.*

The average duration of symptoms before admission to hospital was 4·16 months. Broadbent and Kerman (1951) found that 7·5 months elapsed on average in their series. These two figures are an index of the very real diagnostic difficulties encountered. It may be argued that such a delay is comparable to that encountered in carcinoma of the stomach, a disease far more accessible to the radiologist, but in this interval the balance swings towards inoperability.

Pain was far and away the most frequent initial symptom, occurring in 29 cases (63 per cent), and jaundice a poor challenger with an incidence of only 9 cases (19·5 per cent). General weakness occurred initially in 4 cases (8·7 per cent), anorexia in 3 cases (6·5 per cent), nausea, constipation and diarrhoea each in 2 cases (4·3 per cent). Venous thrombosis in the legs occurred initially in 2 cases (4·3 per cent), and the only case of islet-cell carcinoma in the series presented in coma.

When the total presenting symptoms up to the time of admission to hospital were analysed, a different picture emerged. Pain was still ahead with an incidence of 84·7 per cent and loss of weight (to an average of 23·9 lb.) close behind with a frequency of 80·3 per cent. Jaundice occurred in 67·5 per cent and was painless in only 13 per cent of cases. The other main complaints were anorexia (54·3 per cent), nausea or vomiting (19·5 per cent) and diarrhoea (8·7 per cent).

It is suggested, therefore, that pain is the dominant symptom in pancreatic carcinoma and of great importance as a guide to early diagnosis. Likewise weight loss of considerable degree in the absence of obvious cause should focus thought upon the pancreas. The symptom of jaundice is clearly one of great importance, but the concept of it (especially in its painless form) as the chief and classical sign-post to a diagnosis of carcinoma of the pancreas can be grossly misleading.

An uncommon but important association with this disease is that of psychological disturbances, usually with depressive or apathetic features, and such occurred in 3 cases in the present series. Yaskin (1931) and Latter and Wilbur (1937) have described this association, and it is of special importance to remember it in the presence of undiagnosed abdominal or back pain. Such a patient may appear markedly neurotic, partly, perhaps, because no one seems to understand his complaint, and this can lead to a tragic misdiagnosis in the presence of a painful disease.

*A consideration of pain in pancreatic carcinoma.*

An attempt at correlation between the site of the tumour and the site of pain produced is summarised in Table I.

TABLE I.—*Correlation between Site of Tumour in Pancreas and Site of Pain.*

| | Back (per cent). | Epigastric (per cent). | Right upper (per cent). | Left upper (per cent). | Lower (per cent). |
|---|---|---|---|---|---|
| Of 31 cases involving the head | 25·8 | 45·2 | 38·7 | 12·9 | 6·5 |
| Of 14 cases involving body, tail or both | 64·3 | 78·5 | 18·0 | 18·0 | 21·4 |
| Of 4 cases involving tail alone | 75·0 | 75·0 | 25·0 | 0 | 50·0 |

Of 7 cases presenting without pain, the growth involved the head in 6, extending to the body in 2. The seventh, an islet cell carcinoma of the tail, presented in coma, and no clear history was obtainable. Excluding this latter case, every tumour involving the whole gland, the body alone, the body and tail, and tail alone gave rise to pain.

The incidence of right upper quadrant pain was higher in tumours involving the head. Epigastric and back pain occurred more commonly in growths involv- the body and tail. Left upper quadrant pain was relatively uncommon in any group, and growths involving the tail alone showed a marked tendency to give pain referred to the lower abdomen.

The postural relief and aggravation of pain as described by Da Costa (1858), was a marked feature in 8 cases, and in 4 cases pain of a peptic ulcer type, relieved by food and alkalis, occurred.

*Abnormal physical signs.*

Clinical jaundice was noted in 28 cases (60·8 per cent) on admission, and of these 17 cases had demonstrable hepatic enlargement, and 6 a definitely palpable gall-bladder. Three cases giving a history of recent jaundice were not noted to have clinical icterus on admission.

Of 19 non-jaundiced patients, only 4 had clinical enlargement of the liver, and none a definitely palpable gall-bladder.

Liver enlargement ranged from 3 to 6 finger breadths below the costal margin.

In 19 cases a mass considered to be the pancreatic tumour was palpable, and abdominal tenderness was noted in 14. One case had active thrombo-phlebitis, and 1 presented with paraplegia due to extradural metastases.

In many cases the disease was well-advanced by the time that abnormal signs were clear cut. The discovery at operation of a dilated gall-bladder that had defied clinical palpation occurred on several occasions.

*Investigations of particular value.*

1. *Radiological study with barium meal.*—Of 26 cases examined, a definite diagnosis was made in 11 (42 per cent), and some abnormality detected in 12 others (46 per cent). Abnormalities of stomach contour were noted in 11 cases. Deformity of the antrum with indentation on either curvature seen in the antero-posterior view (Fig. 1), and indentation of the posterior stomach wall seen in the lateral view (Fig. 2) were helpful features.

Abnormalities of contour or mucosal pattern occurred in the duodenal cap in 10 cases, and in the duodenal loop in 9. An example of widening of the duodenal loop is shown in Fig. 1. This area is generally recognised as one yielding classical diagnostic features, but it is also important to recognise early and minor changes. Fig. 3*a* and *b* illustrate a case in which a small abnormal segment at the beginning of the duodenal loop gave way to gross changes of disorganisation in the interval of 20 months elapsing between examinations (*a*) and (*b*).

In 3 cases changes were found in the 3rd and 4th parts of the duodenum. Fig. 4 illustrates a case of carcinoma involving the tail and body of the gland with indentation of the upper border of the 3rd part of the duodenum. Another helpful finding in 2 cases of tumour in this site was forward displacement of the duodeno-jejunal flexure. It is of interest that Broadbent and Kerman (1951) increased the percentage of their cases diagnosable on radiological grounds from 18·4 per cent to 53·9 per cent on retrospective review of the films. It is felt that the trained radiologist can often make the major contribution towards diagnosis.

2. *Tests of carbohydrate metabolism.*—Three cases in this series were known diabetics of many years standing. Four cases had diabetes diagnosed within 8 months of admission to hospital, and in these cases the diabetes may have been a manifestation of the progressive pancreatic lesion.

Repeated glycosuria occurred in 14 (32·2 per cent) of the total 46 cases. The fasting blood sugar was raised above 110 mg. per cent in 20 of 40 cases (50 per cent). Of 8 cases examined by the glucose tolerance test, a diabetic type curve was obtained in 6 cases (75 per cent). These latter cases included 2 of the known cases of diabetes of recent onset, but none of long standing. Two cases with normal fasting blood sugar and no glycosuria gave a diabetic type response to the glucose tolerance test. Dashiell and Palmer (1948) obtained a diabetic type curve in 85·7 per cent of 21 cases examined.

It is suggested that the glucose tolerance test is of great importance in the diagnosis of carcinoma of the pancreas, and that minor or moderate degrees of impairment of carbohydrate metabolism may escape detection by any less demanding test.

3. *Serum enzyme determinations.*—Serum lipase determinations (by the Loerenhart method modified by Cherry and Crandell, 1932) in 37 cases gave a value

---

EXPLANATION OF PLATE.

FIG. 1.—Indentation of the antrum of the stomach on the greater curvature, flattening of the greater recess of the duodenum, and widening of the duodenal loop in a case of carcinoma of the head of the pancreas.

FIG. 2.—This illustrates the value of the lateral view of the stomach in demonstrating indentation of the posterior wall by pancreatic growth.

FIG. 3.—This shows the significance of minor changes in the contour and mucosal pattern of the duodenum. 20 months elapsed between examination (*a*) and (*b*).

FIG. 4.—A case of carcinoma of the tail and body of the pancreas producing indentation of the upper border of the 3rd part of the duodenum.

1

2

3a

3b

4

exceeding 1·0 c.c. of N/20 sodium hydroxide in 15 cases (40·5 per cent). Comfort and Osterberger (1940) reported an identical percentage positivity in their cases using a figure of 1·5 c.c. N/20 sodium hydroxide as the upper limit of normality. Johnson and Bockus (1940) reported a raised value in 5 of 8 cases.

Secretin stimulation of the pancreas was performed in 3 cases, accentuating an already raised level in 2 cases, and producing an abnormal value in 1. Of 27 jaundiced cases the serum lipase was raised in 12 and normal in 15, indicating that this test is independent of biliary obstruction.

The serum amylase was raised above 125 Somogyi units in 7 of 34 cases (20·6 per cent). On secretin stimulation a raised value was produced in 3 of 4 other cases. The amylase determination is therefore considered to be of less value than the lipase.

4. *The bromosulphalein retention test* was performed in 17 cases, all of which showed abnormal dye retention.

Of these cases, 9 had hyperbilirubinaemia, of which 3 were subsequently shown to have liver metastases. Five cases without hyperbilirubinaemia all had liver metastases at operation.

It is suggested that this test may be of value in indicating multiple liver metastates in the absence of biliary obstruction.

*Other investigations reviewed.*

The serum bilirubin was raised above 1 mg. per cent in 27 of 33 cases investigated, of which 24 had clinical jaundice.

The serum alkaline phosphatase was examined in 26 cases and was raised above 5 Bodansky units in 13, of which all had associated hyperbilirubinaemia. Of 13 cases shown to have liver metastases, the alkaline phosphatase was raised in 7 and normal in 6.

The serum cholesterol was raised above 220 mg. per cent in 20 of 26 cases, and hyperbilirubinaemia accompanied 18 of these 20 cases. A normal chloesterol level occurred in 5 cases with associated hyperbilirubinaemia. The cholesterolesters exceeded 50 per cent of the total cholesterol in 20 of 21 cases.

It appears, therefore, that these latter three investigations reflect only the presence of biliary obstruction, and beyond this are not helpful in the present diagnostic problem.

The plasma proteins were normal in 23 of 25 cases, there being some degree of hypoalbuminaemia in the remaining 2 cases.

The cephalin, thymol and colloidal gold flocculation and turbidity tests were normal in all of 22 cases examined. In 3 cases the cephalin flocculations test became positive after cholecysto-enterostomy. The value of these tests in the present connection is that they facilitate exclusion of hepatitis in cases presenting with jaundice, as pointed out by Maclagan (1947)

Of 46 cases examined, 31 (67·4 per cent) had haemoglobin levels below 13·0 g. per cent, with an average of 12·13 (76 per cent Hb.) for the whole series. The red blood cell counts ranged from 3·19 to 5·6 millions, with an average of 4·29 millions. No profound anaemia occurred.

The mild degree of anaemia encountered suggests that this feature may be valuable in the differential diagnosis from other forms of intra-abdominal malignancy.

DISCUSSION.

In view of the inherent difficulty of diagnosis of carcinoma of the pancreas at a stage where surgical resection is possible, it is necessary that all means of investigation should be combined at the earliest opportunity.

Firstly the clinician must be alert to the modes of presentation of the disease and its substantial, and possibly increasing, incidence in the community. Once clinical suspicion has been aroused, a combined plan of investigation, including careful radiological study, a glucose tolerance test, serum enzyme determinations, and possibly a bromsulphalein retention test, offers considerable chance of providing evidence in support of such suspicion. Bourne (1936) has also advocated a stool fat analysis, and this might well be included.

The cytological examination of duodenal contents as reported by Lemon (1951, 1952) and Rubin, Palmer and Kirsner (1952) gives promise of further valuable help in diagnosis.

Should all investigations prove unrewarding and a strong clinical suspicion still remain, it would appear justifiable to proceed to surgical exploration, as has been advocated by Bourne (1936).

SUMMARY.

The historical background of carcinoma of the pancreas is briefly reviewed, A series of 46 cases proven at operation is considered from the clinical, radiological and biochemical aspects in an attempt to clarify the helpful diagnostic features. The frequent incidence and importance of pain and weight loss as symptoms is emphasized. Jaundice is found to occur less frequently than either of the symptoms mentioned above, and to be painless in relatively few cases. A relationship between the anatomical position of the tumour and the site of the pain complained of by the patient is suggested.

A scheme of investigation is proposed which includes careful radiological studies, a glucose tolerance test, serum lipase and amylase determinations and a bromsulphalein retention test. Surgical exploration is advocated where strong clinical grounds alone exist.

I wish to express my warm thanks to Dr. H. L. Bockus, Professor of Gastroenterology in the Graduate Hospital of the University of Pennsylvania, for all his help and encouragement in this investigation. I am also grateful to Dr. A. Finkelstein for the X-ray studies, and to the members of the Medical and Surgical staff of the Graduate Hospital for permission to include cases under their care.

I wish also to thank Sir Stanford Cade for his kind help and criticism.

REFERENCES.

BARD, L. AND PIC, A.—(1888) *Rev. Médecine*, **8**, 257.
BERK, J. E.—(1941) *Arch. intern. Med.*, **68**, 525.
BOURNE, G.—(1936) *Lancet*, ii, 1826.
BROADBENT, T. R. AND KERMAN, H. D.—(1951) *Gastroenterology*, **17**, 163.
CHAUFFARD, M. A.—(1908) *Bull. Acad. Méd., Paris*, **60**, 242.
CHERRY, I. S. AND CRANDELL, L. A.—(1932) *Amer. J. Physiol.*, **100**, 266.
COMFORT, M. W. AND OSTERBERG, A. E.—(1940) *Proc. Mayo Clin.*, **15**, 427.

Da Costa, J. M.—(1858) *N. Amer. Medico-chir. Rev.*, **2**, 883.
Dashiell, G. F. and Palmer, W. L.—(1948) *Arch. intern. Med.*, **81**, 173.
Duff, G. L.—(1939) *Johns Hopk. Hosp. Bull.*, **55**, 69.
Eusterman, G. B.—(1922) *Trans. Amer. gastro-ent. Ass.*, **25**, 26.
*Idem* and Wilbur, D. L.—(1933) *Sth. med. J., Nashville*, **26**, 875.
Finney, J. M. T.—(1910) *Ann. Surg.*, **51**, 818.
Gordon-Taylor, G.—(1934) *Ibid.*, **100**, 206.
Johnson, T. A. and Bockus, H. L.—(1940) *Arch. intern. Med.*, **66**, 62.
Kiefer, E. D.—(1927) *Ibid.*, **40**, 1.
Latter, K. A. and Wilbur, D.—(1937) *Proc. Mayo Clin.*, **12**, 457.
Lemon, H. M.—(1951) *N.Y. St. J. Med.*, ii, 2155.—(1952) *Ann. intern. Med.*, **37**, 525.
Levy, H. and Lichtman, S. S.—(1940) *Ibid.*, **65**, 607.
Maclagan, N. F.—(1947) *Brit. med. J.*, ii, 197.
Mirallie, C.—(1893) *Gaz. Hop. Paris*, **66**, 889.
Mondière, J. T.—(1836) Quoted by Parmentier, E., and Chabrol, E. (1923), *Nouveau Traité de Médecine*, XV, 197.
Rubin, C., Palmer, W. L. and Kirsner, J. B.—(1952) *Gastroenterolojy*, **21**, 1.
Speed, K.—(1920) *Amer. J. med. Sci.*, **160**, 1.
Whipple, A. O.—(1938) *Amer. J. Surg.*, **40**, 260.
*Idem*, Parsons, W. B. and Mullins, C. R.—(1935) *Ann. Surg.*, **102**, 763.
Yaskin, J. C.—(1931) *J. Amer. med. Ass.*, **96**, 1664.